# A Single Disulfide Bond Disruption in the β3 Integrin Subunit Promotes Thiol/Disulfide Exchange, a Molecular Dynamics Study

**DOI:** 10.1371/journal.pone.0059175

**Published:** 2013-03-18

**Authors:** Lihie Levin, Ehud Zelzion, Esther Nachliel, Menachem Gutman, Yossi Tsfadia, Yulia Einav

**Affiliations:** 1 Biochemistry and Molecular Biology Department, George S. Wise Faculty of Life Sciences, Tel Aviv University, Tel Aviv, Israel; 2 Mathematical Biology Unit, Faculty of Sciences, Holon Institute of Technology, Holon, Israel; University of Medicine and Dentistry of New Jersey, New Jersey Medical School, United States of America

## Abstract

The integrins are a family of membrane receptors that attach a cell to its surrounding and play a crucial function in cell signaling. The combination of internal and external stimuli alters a folded non-active state of these proteins to an extended active configuration. The β3 subunit of the platelet αIIbβ3 integrin is made of well-structured domains rich in disulfide bonds. During the activation process some of the disulfides are re-shuffled by a mechanism requiring partial reduction of some of these bonds; any disruption in this mechanism can lead to inherent blood clotting diseases. In the present study we employed Molecular Dynamics simulations for tracing the sequence of structural fluctuations initiated by a single cysteine mutation in the β3 subunit of the receptor. These simulations showed that *in-silico* protein mutants exhibit major conformational deformations leading to possible disulfide exchange reactions. We suggest that any mutation that prevents Cys560 from reacting with one of the Cys^567^–Cys^581^ bonded pair, thus disrupting its ability to participate in a disulfide exchange reaction, will damage the activation mechanism of the integrin. This suggestion is in full agreement with previously published experiments. Furthermore, we suggest that rearrangement of disulfide bonds could be a part of a natural cascade of thiol/disulfide exchange reactions in the αIIbβ3 integrin, which are essential for the native activation process.

## Introduction

Integrins are a broad family of membrane-associated heterodimers that mediate any interaction between a cell and its surrounding tissues. The non-covalently associated trans-membrane α and β integrin subunits contain both small intracellular and very large extracellular parts. The human platelet integrin αIIbβ3, also termed glycoprotein IIb/IIIa, is a typical representative of this wide family of cell adhesion receptors [1]. αIIbβ3 plays a crucial role in mediating platelet adhesion and aggregation by serving as a fibrinogen and a von Willebrand factor receptor. The binding of ligands to αIIbβ3 is strongly regulated; following activation by inside-out signals, the integrin goes through conformational changes resulting in ligand binding, clustering of the αIIbβ3 receptors, tyrosine phosphorylation and cytoskeleton rearrangements [2], [3]. The precise mechanism of αIIbβ3 activation is still unknown, but the crystallographic data suggest equilibrium between a bent and an extended conformation, which is able to bind a ligand. Several intermediate affinity states have also been suggested [4]–[7], and an alternative model involving release of a “deadbolt” created by an interface of a two distant domains has also been proposed [8]–[10].

Both αIIb and β3 subunits of the protein contain many conserved cysteine residues, which form disulfide bonds. The extracellular β3 subunit contains 56 highly conserved cysteines located in the four integrin-epidermal growth factor-like (I-EGF) repeats and a β-tail domain, situated in the membrane-proximal, “lower leg” region of the protein. Several studies have shown that the integrin activation process depends on the formation of free thiols with a subsequent disulfide exchange reaction [11]–[14]. Indeed, addition of dithiothreitol, a reducing agent, leads to global conformational changes and the opening of ligand binding site [15]. Integrin αIIbβ3 has also been shown to exhibit endogenous thiol isomerase activity [16]. Moreover, the active state of the integrin contains more free thiols than the resting conformation [17]. Therefore, the cysteine-rich region of β3, which is located between the cytoplasmic domains and the ligand-binding headpiece, is purported to be the fulcrum of rearrangement events which are associated with the activation [18]. In accordance with this assumption, there are several natural mutations in cysteine residues that lead to the inherited bleeding disorder Glanzmann Thrombasthenia (GT), displaying a defective platelet aggregation. The phenotypes of the various mutations associated with this disorder range from the total absence of αIIbβ3 on the platelet membrane (due to abnormal conformation of β3) to a constitutively active complex permanently locked in a high-affinity state [19]–[22]. Interestingly, some cysteine mutations in the same β3 subunit affect the conformation and function of the αIIbβ3 and the closely related αvβ3 complexes differently [23]. How the disulfide isomerization and the free thiols integrate into the integrin activation models is still unresolved and the specific redox sites in the β3 integrin subunit have not yet been described.

Each of the four I-EGF domains located in the “lower leg” of the β3 subunit contain eight cysteines which, according to crystal structures, form four disulfide bonds in an order of: 1–5, 2–4, 3–6 and 7–8 pattern (except for I-EGF1 that lacks the 2–4 bond) [24], [25]. The 1–5 bond is unique for the EGF element of the integrins, whereas the 2–4, 3–6 and 7–8 bonds are conserved in other EGF domains [18], [26]. It should be noted that, according to a trypsin digestion assay, the pattern of pairing was slightly different, e.g. for the I-EGF4 domain, the 1–2 and 3–5 pairing were proposed [27]. Thus, it might be possible that under *in vivo* conditions the disulfide pattern is flexible and varies according to the physiologic state. Considering that for all published crystal structures of the complete ectodomain [6], [24], [25], [28] the integrins are proposed to be in their non-active state, it is possible that the active form of the receptor may contain free thiols and that disulfide rearrangement is an integral process of coupling the cytoplasmic tails with the large extracellular headpiece during integrin activation.

**Figure 1:**
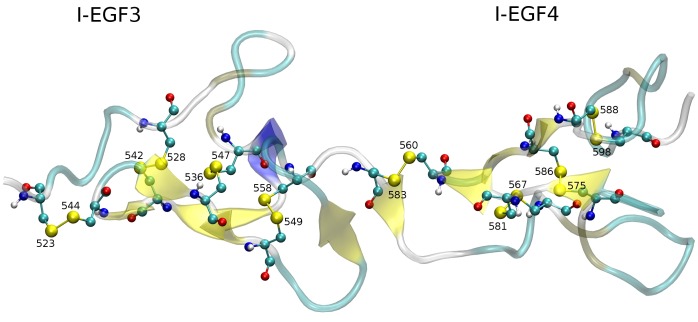
Disulfide network in I-EGF3 and I-EGF4. The figure is taken from the crystal structure, PDB code: 3FCS (*27*). Domains I-EGF3 and I-EGF4 are in cartoon representation; Cysteine residues are shown in balls-and-sticks and numbered as in the mature protein.

In this study we sought to investigate the outcome of disulfide bond opening by *in-silico* mutation of one of its cysteine residues using Molecular Dynamics (MD) simulations. The Cys^560^–Cys^583^ bond is the integrin-specific 1–5 pair located in the I-EGF4 domain [18], [26]. The natural occurring mutation C560R was identified as the cause for the GT bleeding disorder and is coupled with a constitutively active state of the αIIbβ3 integrin. On the other hand, the artificial mutation, in which the other cysteine of the pair (Fig. 1) is replaced by serine (C583S), displays an almost normal state of activation [22]–[24]. Another important pair is the Cys^567^–Cys^581^ bond (2–4 pair), in which replacement of either the Cys567 or the Cys581 residues blocks the activation of the mutated protein [29]. In the present study we shall address the possibility that opening of such a disulfide bond may initiate structural rearrangements leading to the approach of a free thiol to an existing disulfide with subsequent disulfide isomerization. The question associated with such a reaction is whether the sequence of thiol/disulfide exchange events will be random or, the internal structure of the protein will impose a predetermined sequence of structural rearrangements, leading to regiospecificity.

The MD simulation method has been used successfully in several investigations of the αIIbβ3 structure [30]–[34]. However, these studies focused mainly on the headpiece region and the ligand-binding site. In the present study we investigate a single disulfide bond disruption in the “lower leg” region of the β3 subunit. We show structural consequence of bond opening and demonstrate that in some cases it enables a thiol/disulfide exchange reaction of the free thiol with another cysteine pair. An artificial exchange reaction, followed by additional MD simulations, reveals proteins that strongly prefer defined conformational states. To our best knowledge, this is the first time that specific thiol/disulfide exchange events in the αIIbβ3 integrin have been projected by computational technology.

## Materials and Methods

### Molecular Dynamics Simulations

The atomic coordinates of the I-EFG1, I-EFG2, I-EFG3, I-EFG4 and β-tail fragment (β3 subunit residues: 436–690) of integrin αIIbβ3 were obtained from the Protein Data Bank (PDB ID: 3FCS) [25]. Completion of the six missing residues (477–482) in the 3FCS protein structure and implementation of the point mutations (C583S, C560R and C581S) were performed using the Swiss-PDB Viewer program [35].

Table 1 presents a list of the simulations discussed in our study. The wild type 1 (WT-1), C583S and C560R MD simulations were performed using GROMACS 3.3.3 suite of software [36], whereas the disulfide exchange MD simulations (Cys^560^–Cys^567^, Cys^560^–Cys^581^ and Cys^567^–Cys^586^, WT-2 and the C581S) were performed using GROMACS 4.07 [37]. All of the simulations were carried out using the GROMACS 53a6 force field [38].

**Table 1:** List of simulations of integrin αIIbβ3, discussed in the present study.

	Simulation	Free Thiol	Total Simulation time (ns)	Simulation Type
1	WT	None	70^a^	Wild type protein
2	C560R	Cys583-SH	35	*In silico* mutation
3	C583S	Cys560-SH	341^a^	*In silico* mutation
4	Cys^560^–Cys^567^	Cys581-SH	35	*In silico* thiol/disulfide exchangeproduct of C583S mutation
5	Cys^560^–Cys^581^	Cys567-SH	35	*In silico* thiol/disulfide exchangeproduct of C583S mutation
6	C581S	Cys567-SH	90^a^	*In silico* mutation
7	Cys^567^–Cys^586^	Cys575-SH	40	*In silico* thiol/disulfide exchangeproduct of C581S mutation

a 2 simulations of WT protein, 7 of C583S (with different restrain conditions; for more details see Table S1) and 3 of C581S were conducted.

The proteins were immersed in triclinic boxes filled with SPC [39] water molecules that extended to at least 12 Å between the molecule and the edge of the box. Na^+^ and Cl^-^ ions were added to neutralize the system up to a physiological salt concentration of 100 mM. The whole system - protein, water and ions - was energy-minimized using the steepest descent algorithm with a force tolerance of 1000 kJ mol^−1^ nm^−2^. A 40 ps simulation with position restraints was performed at 300 K to “soak” the water molecules into the protein. The system was simulated further for a 1 ns unrestrained equilibration simulation. Starting with the final structure of the preliminary simulation, the MD simulation was set up. Considering that our simulations were limited to a section of the protein, position restraint was applied on the edges of the protein in order to mimic the natural constraint on these atoms in the intact protein. Varying force intensities were applied between simulations, trying to reach maximal flexibility without losing the basic structure of the protein. The initial force tested in our study was 1000 kJ mol^−1^ nm^−2^ applied on all heavy atoms of the first residue of the N terminus and on all heavy atoms of the last two residues of the protein on the C terminus. Under this force the protein was too rigid, thus in the succeeding simulations the force was applied only on the first heavy atom of I-EGF1 and the last heavy atom of the β-tail domain. Finally, to attain maximal conformational changes we reduced the force applied on the atoms to 100 kJ mol^−1^ nm^−2^; this ensured maximal flexibility while the protein maintained its structure. Detailed information can be found in the supplementary data (Table S1).

The MD simulations were run under NPT (constant Number of particles, Pressure, and Temperature) conditions using Berendsen’s coupling algorithm for keeping the temperature and pressure constant (P = 1 *b*ar; τ_p_ = 0.5 ps; T = 300 K; τ_R_ = 0.1 ps) [40]. During the runs, the LINCS (Linear Constraint Solver) algorithm [41] was used to constrain the lengths of all bonds; the water molecules were restrained using the SETTLE algorithm. A 12 Å cutoff was used for the Van der Waals interactions. The long-range electrostatic interactions were treated by the Particle Mesh Ewald method [42]. The coordinates were saved every 1 or 2 ps. The MD simulation time-step was 2 fs.

### Trajectory Analysis

Trajectories obtained from various simulations were analyzed using flexible tools provided by GROMACS. Visual Molecular Dynamics (VMD) was used for the visual analysis of the trajectories [43].

Cluster analysis was performed for the trajectories of the simulations by the command g_cluster of the GROMACS 4.07 package. Cluster analysis was applied using the Gromos algorithm with an RMSD (Root Mean Square Deviation) cut-off value of 0.4 nm [44].

### Disulfide Exchange

Last frame of C583S simulation was saved and the free sulfur of the Cys560 was connected either to Cys581 or Cys567 and this structure used as an initial coordinate for two other 30 ns simulations, named Cys^560^–Cys^581^ and Cys^560^–Cys^567^, respectively (Table 1). For the Cys^567^–Cys^581^ exchange simulation the last frame of C581S run was saved and the free sulfur of the Cys567 was connected to the Cys586 and simulated for extra 40 ns (Table 1, Cys^567^–Cys^586^).

### Disulfide Pairing in the 3FCS Crystal

WT disulfide pairing in the I-EGF4 domain of the integrin αIIbβ3 as resolved in the 3FCS crystal [25] (Cys^560^–Cys^583^, Cys^567^–Cys^581^, Cys^575^–Cys^586^, Cys^588^–Cys^598^) is detailed in Figure 1.

## Results

### The Structural Fluctuation of the Simulated Proteins

**Figure 2:**
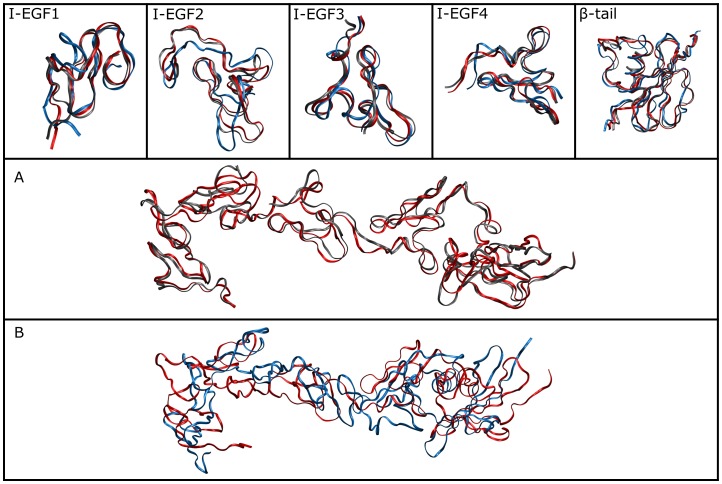
Superposition of the initial and the simulated structures. A - Superposition of the initial structure (red) vs. the most favorable conformation of a simulated WT protein (gray). B - Superposition of the initial structure (red) vs. the most favorable conformation of the simulated C583S mutant (blue). The upper panels introduce the differences in each domain (I-EGF1 to β-tail as indicated); the lower panels show the entire penta-domain structure.

The WT protein and the *in-silico* created C583S mutant of the β3 subunit were subjected to MD simulations for 35 ns. Panels *A* and *B* of Figure 2 show superposition of the starting conformation of the I-EGF1 to β-tail fragment with the most popular conformations of the WT (Fig. 2A) and of C583S (Fig. 2B) accepted along the MD runs. The superposition images of the individual domains are represented in the upper panel of Figure 2. Inspection of the alignment of the starting structure with the simulated WT reveals that the lower part of the β3 subunit maintains a stable structure along the simulation time, both for the individual domains and the entire structure alignments (Fig. 2A and upper panels). This indicates that a simulation of a protein fragment, without introducing any changes to its primary native structure, keeps its conformation at around the starting point. For the C583S mutant, the superposition of the entire structure (Fig. 2B) shows that its global shape was grossly affected by the single mutation. However, no significant alterations in the rigid domain conformations were observed (Fig. 2 upper panels). Thus, the differences between the various structures sampled during the simulation are due to inter-domain movements.

In the C560R mutation, a phenotype characterized by over activation, a significant movement between the domains can be seen when aligning the entire structure. Yet, in contrast with the trajectory of C583S, in the case of C560R there is also a slight motion within the domains (Fig. S1).

The RMSD values for WT, C583S and also for C560R simulations are shown in Figure S2 in the Supporting Material.

**The Distances between Free Thiols and Disulfide Bonds**

The Cys^560^–Cys^583^ bond is positioned near the junction between I-EGF3 and I-EGF4. Thus, two potential disulfide exchange events are optional; the first is with the Cys^549^–Cys^558^ pair located in I-EGF3 and the second is with the Cys^567^–Cys^581^ pair located in I-EGF4. To check the possibility of disulfide isomerization in cysteine mutants, we monitored the distance between the free cysteine (Cys583-SH or Cys560-SH) and the closest disulfide bonds during the simulation (Table 1, simulations 2 and 3).

**Figure 3:**
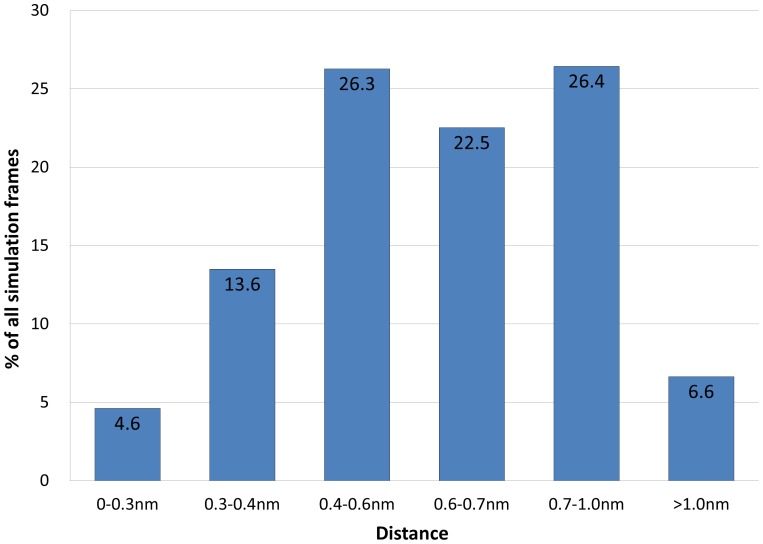
Distance range between a free thiol of Cys560 and a Cys567–Cys581 bond. The minimum distance between Cys560 free thiol and Cys567–Cys581 pair was calculated in all the seven C583S simulations. The number of frames in which the distance lies in a certain range was computed over a total of ∼188,000 simulation frames and is presented inside the columns.

Seven independent simulations of the *“in-silico”* C583S mutated protein, (Table S1, total 341 ns, 188,000 frames) were analyzed and the number of frames in which the free thiol of Cys560 was within a certain distance range from the nearest disulfide bond was calculated (Fig. 3). In 18.2% of the total simulation frames, the free thiol of Cys560 was in very close proximity (<0.4 nm) to the Cys^567^–Cys^581^ bond and in an additional 26.3% of the frames, the distance was within a range of 0.4–0.6 nm. Overall, in 44.5% of total simulation times the distance between the free thiol and the disulfide bond was shorter than 0.6 nm. Under physiologic conditions, once the sulfhydryl comes within such proximity to a disulfide bond, an exchange event is possible, with subsequent appearance of a new stable structure.

**Figure 4:**
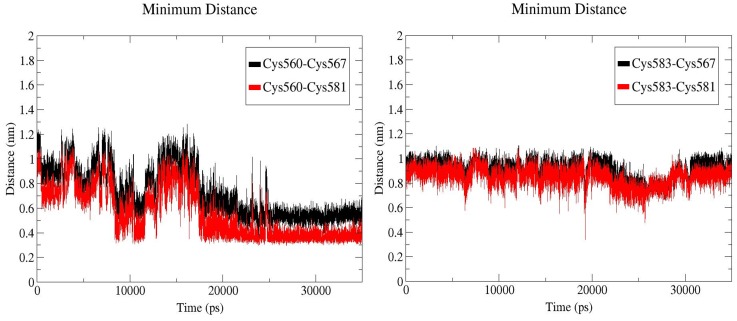
Distance between disulfide pairs as a function of simulation time. Distance between sulfur atoms of the free thiol Cys560 in the representative C583S simulation and of the Cys^567^–Cys^581^ bond (left). Distance between sulfur atoms of the free thiol Cys583 in the C560R simulation and of the Cys^567^–Cys^581^ bond (right).

Figure 4 depicts the variation over time of the distance between the free thiol of Cys560 and the nearby disulfide pair, in a typical C583S simulation (Table S1, number 7). The results revealed a distinct preference for the Cys^567^–Cys^581^ pair (Fig. 4, left panel). At the beginning of the simulation, the free thiol of Cys560 was ∼10 Å from this pair. Yet, within 10 ns, the distance was drastically shortened, and after some fluctuations it finally stabilized at a distance range of 3.5–5.2 Å, until the end of the simulation time.

The naturally occurring C560R mutation is known to constitutively activate integrin. The simulation of this mutant revealed that the free thiol of Cys583 did not approach any of the nearest disulfide bonds and maintained an average distance of 8.5 Å from the Cys^567^–Cys^581^ bond during the entire simulation time (Fig. 4, right panel). Apparently the asymmetric abolishment of a disulfide bond can lead to far-reaching consequences at the physiologic level.

It should be emphasized that in both trajectories (C560R and C583S) the free sulfhydryl groups made no attempt to make contact with any of the disulfide pair of I-EGF3 (see Figure S3). Thus, we can suggest that these mutations do not lead to a merging between the I-EGF3 and I-EGF4 domains of the β3 subunit.

### The Rearrangement of the Disulfide Network

**Figure 5:**
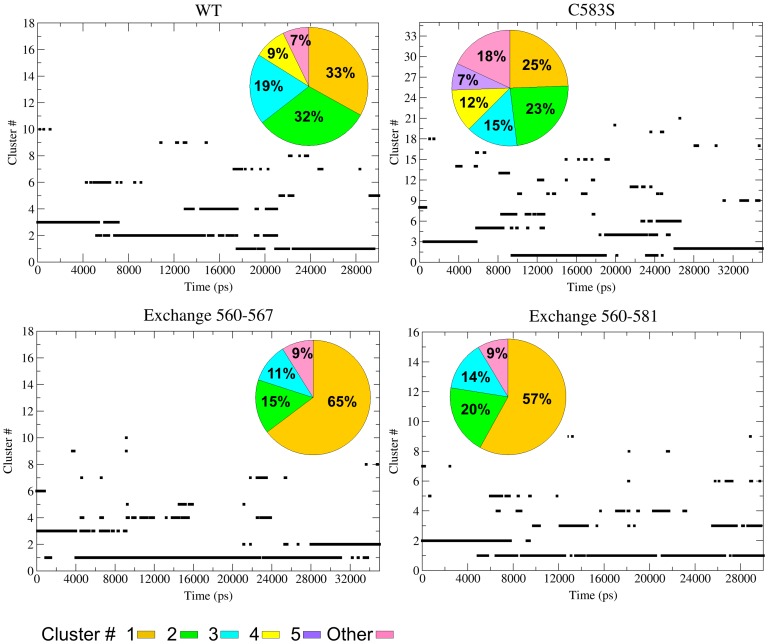
Cluster analysis of WT, C583S and two exchange simulations. The figure depicts the results by two presentations for the four simulations as indicated in each panel. The main panel in each frame shows the transitions between clusters (identified on the ordinate by numbers) along the trajectories. The relative fraction of the dominant clusters is presented by the colored histogram. The clusters are numbered sequentially from the most (#1) to the less popular. The identity of the system under analysis is marked in the frames.

In order to reveal the consequences of disulfide exchange events, the two feasible *in-silico* exchange reactions were generated, and simulated for 35 ns. The free thiol of Cys560 was connected to either Cys567 or to Cys581, making new Cys^560^–Cys^567^ or Cys^560^–Cys^581^ disulfide bonds for the two consequent MD simulations (simulations 4 and 5, Table 1). Generally, the outcome of the simulations is not monolithic and the quantitative evaluation of the various structures is needed. To characterize the many conformational states sampled by the simulations of the WT, C583S and the two exchange products (Cys^560^–Cys^567^ and Cys^560^–Cys^581^), cluster analysis was performed (Fig. 5).

The cluster analysis was carried out with a cut-off value of 0.4 nm, a value that generated more than 10 clusters per simulation, each containing a sufficient number of configurations to attain statistical significance. The main panels in Figure 5 depict the sequence of the structural fluctuations that the protein undergoes, classifying the momentary structure according to the cluster type. Thus, in the WT trajectory the initial state corresponded with cluster #3, with a brief sampling of the configurations corresponding with clusters #10 and #6. After ∼6 ns, cluster #2 became more prevalent and at ∼18 ns cluster # 1 became the most popular. However, during the whole length of the trajectory the structure gained conformations compatible with the less popular clusters, as demonstrated by the sporadic association with clusters having a lower probability. The overall distribution between the clusters is given by the histogram inserted into the frame. Apparently, the WT protein has 4 major structures that consist of 93% of all configurations, where the difference in free energy between them (calculated as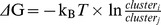) is well below 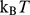. Based on this value, we can assume that all structures are in equilibrium with rapid transitions between them.

The simulation of the C583S mutant reveals slightly higher fluctuations between clusters, as the two largest clusters are almost equal in size and the fraction of the 4 major clusters decreases from 93%, as in the WT, to 75% in C583S (Fig. 5, upper panels). Once the Cys560 is attached to either Cys567 or the Cys581, the protein gains relative stability (with respect to C583S where Cys560 has free thiol) and the largest cluster in both mutants is populated for 65% and 57% of the trajectories, respectively (Fig. 5, lower panels). These results clearly indicate that the disulfide exchange of the Cys560 free thiol with the Cys^567^–Cys^581^ bond stabilizes the protein in a specific structure without preventing it from sampling other conformations.

### The C581S Mutation and the Derived Disulfide Exchange Reaction

As reported previously, any mutation in one of the cysteine residues in the native pair Cys^567^–Cys^581^ significantly reduces the activation of integrin; thus, it was suggested that this disulfide bond makes a structural contribution to the ability of the protein to function normally in the activation mechanism. Moreover, the C581S mutation resulted not only in reduced expression of the integrin, but, more importantly, in a totally inactive status [29]. For that reason it was interesting to mutate C581 to serine *in-silico*, leaving its Cys567 partner with a free SH group (Table 1, number 6), and to examine what structural conformation the protein undergoes, besides the fact that a disulfide exchange with Cys560 is no longer possible.

**Figure 6:**
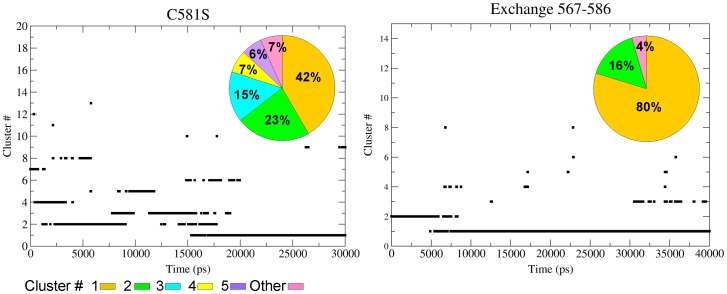
Cluster analysis of C581S and Cys^567^–Cys^586^ simulations. Cluster analysis of C581S (left) and Cys^567^–Cys^586^ (right) disulfide simulations. The figure depicts the results by two presentations for the two representative simulations as indicated in each panel. The main frame shows the transitions between clusters (identified on the ordinate by numbers) along the trajectories. The relative fraction of the dominant clusters is presented by the colored histogram. The clusters are numbered sequentially from the most (#1) to the less popular. The identity of the system under analysis is marked in the frames.

According to the crystal state, residue Cys567 is only ∼4.5 Å from the Cys^575^–Cys^586^ disulfide pair (Figure 1), suggesting that a disulfide exchange is feasible. During the C581S simulations the distances between sulfur atoms of Cys567 from Cys575 or Cys586 were maintained with an average value of 3.7 Å (see Figure S4). None of the other cysteine residues came to a covalent distance with Cys567. This suggests that the free Cys567 residue can also participate in, or initiate, a set of disulfide rearrangements, where the Cys^575^–Cys^586^ bond is the preferred target. To check the structural effect of this event we connected the free thiol of Cys567 to the sulfur atom of Cys586 and initiated the simulation from the last snapshots of C581S simulation. The outcome of cluster analysis of C581S mutant and Cys^567^–Cys^586^ exchange, simulated for an additional 40 ns, is presented in Figure 6 and in Fig. S5.

For the C581S mutant, the trajectory is characterized by a dominance of the first and the second cluster, which together consist of ∼2/3 of the total states. Once the free cysteine (Cys567) was linked with Cys586 (the nearest member of the Cys575–Cys586 pair) a very stable structure emerged, where the first cluster was almost 1.6 *k*_B_T more stable than the second (Fig. 6). In comparison with the other simulations described in this study, this mutant exhibits a clear preferential convergence of structure. The trajectory revealed a distinctly dominant structure, generated during the early phase of the simulations, persisting (80% of the total number of states) until the simulation was terminated. These data indicate that in the C581S mutant the thiol/disulfide exchange event locks the protein in one defined conformational space.

## Discussion

Disulfide isomerization *in vivo* produces structural isomers with the same oxidation state; thus, traditional biochemistry techniques have serious limitations in exposing specific redox sites. These approaches usually freeze thiol/disulfide exchange reactions at specific times and analyze the products by high-performance liquid chromatography, electrophoresis or NMR spectroscopy [45], [46]. Recently, a single-molecule force spectroscopy was used to monitor disulfide bond reduction and isomerization [47], [48]. However, the experimental results are frequently difficult to interpret and specific cysteine residues, prone to such an exchange, remain to be defined unambiguously. Therefore, direct identification of thiol/disulfide exchange events in complex proteins and, specifically, in the αIIbβ3 is currently possible only with computational methods, the first attempt of which is presented here.

The lower leg of the β3 subunit is built of a sequence of rigid elements: the four I-EGF domains and the β-tail are all β-sheet structures that are stabilized by internal disulfide bonds. Despite the apparent stability of the structural elements, the αIIbβ3 receptor experiences a massive structural fluctuation during its activation. The presence of multiple disulfides render the protein to be very rigid and resistant to deformation [49]. Thus, the introduction of free thiols that can initiate a sequence of SH/S-S replacements and lead to structural modulation seems to be necessary. In the present study we evaluated the possibility of disulfide bond exchange and to what extent the rearrangement of the disulfide network can contribute to the flexibility and the stability of the protein. For this purpose we introduced *in-silico* mutations representing well experimentally documented mutants, in which a single cysteine of a disulfide bond was replaced either by serine (C583S and C581S) or by arginine (C560R) and monitored the proximity of the partner-free thiol to the other cysteine pairs by MD. Whenever sulfur atoms came within a range where a covalent bond may be formed, we performed an *in-silico* thiol/disulfide exchange and simulated these new structures, focusing on the structural effects of these rearrangements.

The starting point of the study was the Cys^560^–Cys^583^ bond, which experimentally leads to two, antipodal scenarios; either a constitutively active protein (C560R) or an almost normal activation status with reduced expression (C583S) [22], [23], [29]. This indicates that the single disruption of a disulfide bond could lead to two different structural deformations. We allowed both mutants to exercise an unbiased MD run, in the presence of explicit solvent and physiologic ionic strength, focusing on the structural impact of these mutations. Comparison of the trajectory with that of the WT protein indicated that during the simulation, the C583S mutant experienced a massive conformational change. However, none of the domains were distorted, indicating that the main conformational changes are an outcome of inter-domain motions, whereas the sub-elements retained their rigidity. It is of interest to point out that the mechanical stability of multi-domain proteins exhibits a lower resistance to relative motion of the domains compared to deformation of the domains themselves [49].

A mutation of a cysteine residue, which participates in a disulfide bond, not only breaks the link between two cysteine residues but also releases the second cysteine, so it can participate in disulfide exchange reaction with other disulfide bonds that it encounters during structural fluctuation. In the C583S simulation, the sulfhydryl moiety of Cys560, released from the disulfide bond, was free to move around its immediate vicinity. Based on the crystal structure, Cys560 has two possible partners for disulfide exchange; Cys^567^–Cys^581^ or Cys^549^–Cys^558^ which are located at almost equal proximity (Fig. 1). However, the unbound Cys560 did not scan the whole space nearby but tended to favor a certain disulfide, the Cys^567^–Cys^581^ bond (Fig. 4, left panel, Fig. S3). In about 44% of the total number of frames, from 7 different simulations, the free thiol group approached the sulfur atoms, to such proximity that can allow attraction forces to eventually produce an exchange reaction. This region-specificity is probably imposed by the rigidity of the domain gained by the high content of β-strands in this protein region, which limits the ability of the structure to sample a large conformation landscape. In contrast with the scenario observed for the C583S simulation, the simulation of the C560R mutant failed to converge into a stable structure where the Cys583 moiety did not initiate any attack on any other disulfide pair. It should be emphasized that the constitutive activation of the protein, caused by the mutation C560R, is not specific to arginine substitution, it is common to 11 other amino acid replacements of Cys560 [50]. Thus, simulation of C560R should exhibit a similar result as with the other mutations, which can also imply for the insignificance of the side-chain property in this critical condition. As mentioned above, it should also be stressed that *in-vitro* experiments done by Mor-Cohen et al. [23], [29] demonstrated that, in contradiction to the constitutively active Cys560 substitutions, the C583S mutation has nearly normal activation status. By combining these *in vitro* results with the MD simulations performed in this study, it can be suggested that the mutated C583S integrin is spared from gaining the permanent over-activated status, due to conformational changes initiated by the attack of the unbound free Cys560 to the Cys^567^–Cys^581^ pair, thus enabling a thiol/disulfide exchange which alters the rearrangement of disulfide bonds.

In accordance with the established role of disulfide bond reduction during integrin activation, we reduced the Cys^567^–Cys^581^ disulfide bond and connected the free Cys560 to either Cys567 or Cys581. Both structures, containing either a Cys^560^–Cys^567^ or Cys^560^–Cys^581^ bond, were subjected to further MD simulations. Once the new disulfide bonds were formed, the protein acquired new conformations that were evaluated by their relative stability using cluster analysis of the trajectories. In all cases we could identify a dominant structure which was in very rapid equilibrium with other states. This flexibility could lead to other disulfide rearrangements in nature and be the key to the integrin activation process. The rearrangements between Cys^560^–Cys^583^ and Cys^567^–Cys^581^ bonds, tested by MD in this study, are also in agreement with an alternative disulfide pairing of αIIbβ3 suggested by protein digestion assay, which connects Cys560 to Cys567 [27], though this proposed Cys^560^–Cys^567^ bond was not further confirmed by protein crystallization studies. Thus, we suggest that the Cys^560^–Cys^567^ is not a permanent bond, but may be one phase in the multistage activation process of the solvated αIIbβ3 protein.

In the crystal structure the Cys^567^–Cys^581^ and the Cys^575^–Cys^586^ bonds are located in almost covalent distance from each other. To check whether this proximity would persist in a dynamic system, we simulated C581S mutant and monitored the distance between the created free Cys567 to other disulfide bonds. Not surprisingly, the proximity to the Cys^575^–Cys^586^ bond was maintained and even increased. To follow this tendency we performed a thiol/disulfide exchange reaction by linking free Cys567 to Cys586. The resulting product strongly prefers one defined conformational state, spending 80% of simulation time in a specific structure, thereby losing the sampling ability of other conformations. Due to the native proximity of the free Cys567 to the Cys^575^–Cys^586^ bond and to the structural consequence of the exchange with Cys586, we propose that the C581S mutation forces the above exchange, thus bringing the protein to a rigid structure, locked in one specified conformation that is not able to proceed to the activation process. This suggestion agrees with the observation that each distortion of the native Cys^567^–Cys^581^ bond significantly reduces the activation of αIIbβ3 [23], [29].

Based on our simulations, we propose that the ability to produce a disulfide exchange reaction between Cys560 (from the Cys^560^–Cys^583^ pair) with Cys567 from the next pair of cysteine residues (Cys^567^–Cys^581^) is one essential step towards normal activity of the protein. Thus, any mutation in Cys560 or in the Cys^567^–Cys^581^ pair that interrupts this important disulfide exchange reaction would cause a severe failure in the activation mechanism of the integrin, as was found experimentally.

Many studies [22], [23], [29], [50]–[52] have shown that specific mutations in the cysteine residues of β3 give rise to a constitutively active receptor. Basing our reasoning on lack of thiol/disulfide exchange ability, as exemplified by the C560R, we could hypothesize that at least some of these mutations produce a rigid structure, not capable of rearrangement, which is, therefore, fixed in a constitutively active state.

It is clear that thiol/disulfide exchange plays an important role in αIIbβ3 and the closely related α_V_β3 activation and, indeed, unpaired cysteine thiols appear in the β3 subunit of these integrins upon activation [53], [54]. Considering the large number of disulfide bonds, the number of resulting conformations can increase exponentially [47]. However, as presented in this study, the outcome of disulfide rupture is similar to a collapse of domino blocks, following a given pattern. Apparently, the initiation of a single sulfhydryl moiety can generate a unique sequence of sulfhydryl exchange events leading to new stable conformations, one after the other, and resulting in a gradual distortion of the lower leg section of the β3 subunit of the integrin. The cysteine residues investigated in this study are so close on the primary sequence of the protein that the β-sheet structures need to pucker to accommodate the disulfides. This puckering is believed to cause a significant strain on the bonds [55], [56]. There is evidence that locally tolerated stress is important for protein function and that high torsional energies have an enhanced likelihood of reduction [57]. Therefore, the described cysteine residues may have a functional redox role. Recent research has shown that surface thiols in αIIbβ3 are exposed during platelet activation and, more importantly, different populations of the integrin exist on platelets based on differential labeling of thiols [31], [58]. These allosteric thiols may participate in the thiol/disulfide exchange reactions, leading to the integrin activation or deactivation processes. Although crystallography has not identified free thiols in αIIbβ3, it is possible that their oxidation to disulfide bonds occurs in the expression, purification or crystallization procedures.

### Conclusions

The results presented in this study demonstrate the possibility of disulfide exchange in the dynamic system of cysteine mutants of αIIbβ3 integrin and show the structural consequences of these replacements. We show that the conformational space that the protein may scan, once a disulfide bond is cleaved, is rather limited and the structural fluctuations tend to converge into a “predestined” conformation. We suggest that overly rigid structures may not be able to go through a thiol/disulfide exchange and, therefore, give rise to an unnatural state of activation, e.g. a constitutively active or inactive protein. We propose that specific redox sites reported in the present study may play a crucial role in the overall conformational changes of αIIbβ3 integrin during its activation and bidirectional signaling.

## Supporting Information

Figure S1 **Superposition of the WT protein and the C560R mutant.** Superposition of the most favorable conformation of a simulated WT protein (red) vs. the most favorable conformation of the simulated C560R mutant (light blue). The upper panels introduce the differences in each domain (I-EGF1 to β-tail as indicated); the lower panel shows the entire penta-domain structure.
(TIF)

Figure S2 **RMSD of the backbone atoms from their initial coordinates as a function of time.** The average and S.D. of the last 20 ns (in red) are presented above every frame. (A) WT, (B) C560R and (C) C583S.
(TIF)

Figure S3 **The minimum distance between the free thiol Cys560 (in the C583S simulation) and the disulfide pair Cys^549^–Cys^558^ in I-EGF3.** The distance was calculated between the sulfur atoms.
(TIF)

Figure S4 **The minimum distance between the free thiol Cys567 and Cys586 in the three C581S simulations.** The distance was calculated between the sulfur atoms.
(TIF)

Figure S5 **Cluster analysis of simulations number 2&3 of the C581S mutation (left & right respectively).** The figure depicts the results by two presentations for the two simulations. The main frame shows the transitions between clusters (identified on the ordinate by numbers) along the trajectories. The relative fraction of the dominant clusters is presented by the colored histogram. The clusters are numbered sequentially from the most (#1) to the less popular.
(TIF)

Table S1 List of C583S simulations.
(DOCX)
