# Correction: A Single Disulfide Bond Disruption in the β3 Integrin Subunit Promotes Thiol/Disulfide Exchange, a Molecular Dynamics Study

**DOI:** 10.1371/annotation/b4e96e4b-3106-4040-a63c-a3f018f0e5c0

**Published:** 2013-10-16

**Authors:** Lihie Levin, Ehud Zelzion, Esther Nachliel, Menachem Gutman, Yossi Tsfadia, Yulia Einav

The two corresponding authors, Yossi Tsfadia and Yulia Einav, should be noted as contributing equally to this work. 

