# Supplementary figures and images for: A Single Disulfide Bond Disruption in the β3 Integrin Subunit Promotes Thiol/Disulfide Exchange, a Molecular Dynamics Study
Source: PLoS One. 2013 Mar 18;8(3):e59175. doi: 10.1371/journal.pone.0059175 (PMC3601072; doi:10.1371/journal.pone.0059175)

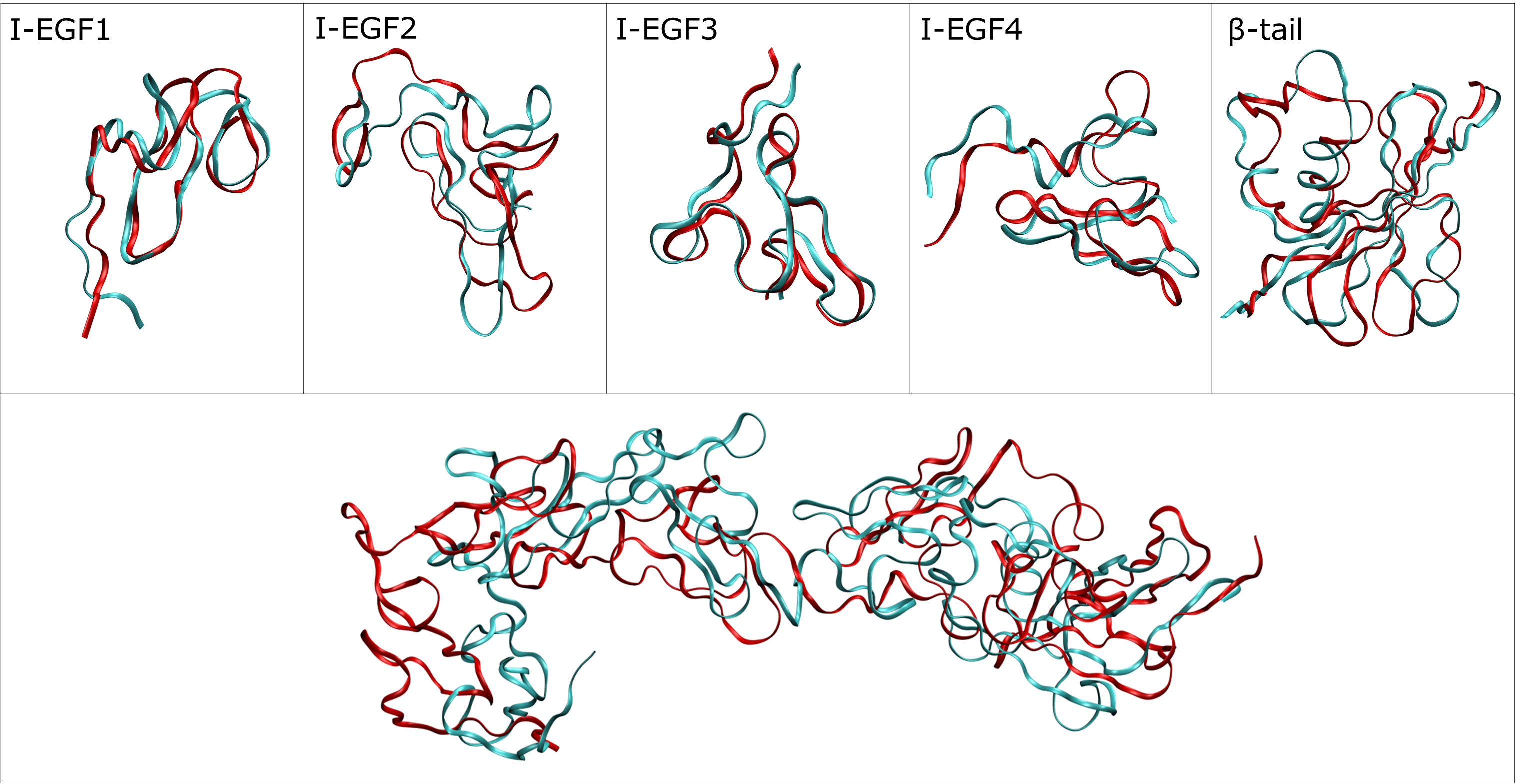

Supplement: Figure S1 — Superposition of the WT protein and the C560R mutant. Superposition of the most favorable conformation of a simulated WT protein (red) vs. the most favorable conformation of the simulated C560R mutant (light blue). The upper panels introduce the differences in each domain (I-EGF1 to β-tail as indicated); the lower panel shows the entire penta-domain structure. (TIF) [file pone.0059175.s001.tif]

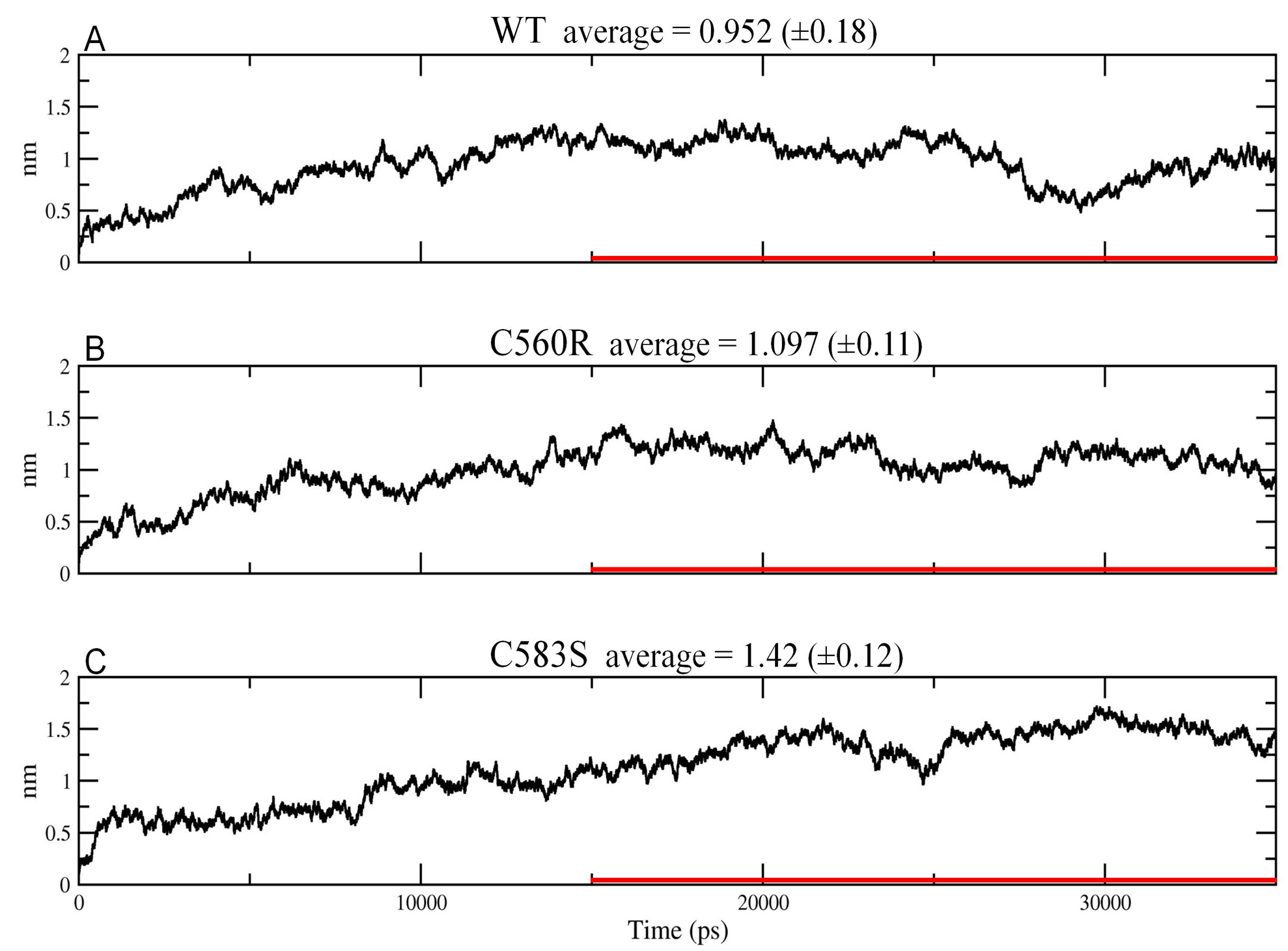

Supplement: Figure S2 — RMSD of the backbone atoms from their initial coordinates as a function of time. The average and S.D. of the last 20 ns (in red) are presented above every frame. (A) WT, (B) C560R and (C) C583S. (TIF) [file pone.0059175.s002.tif]

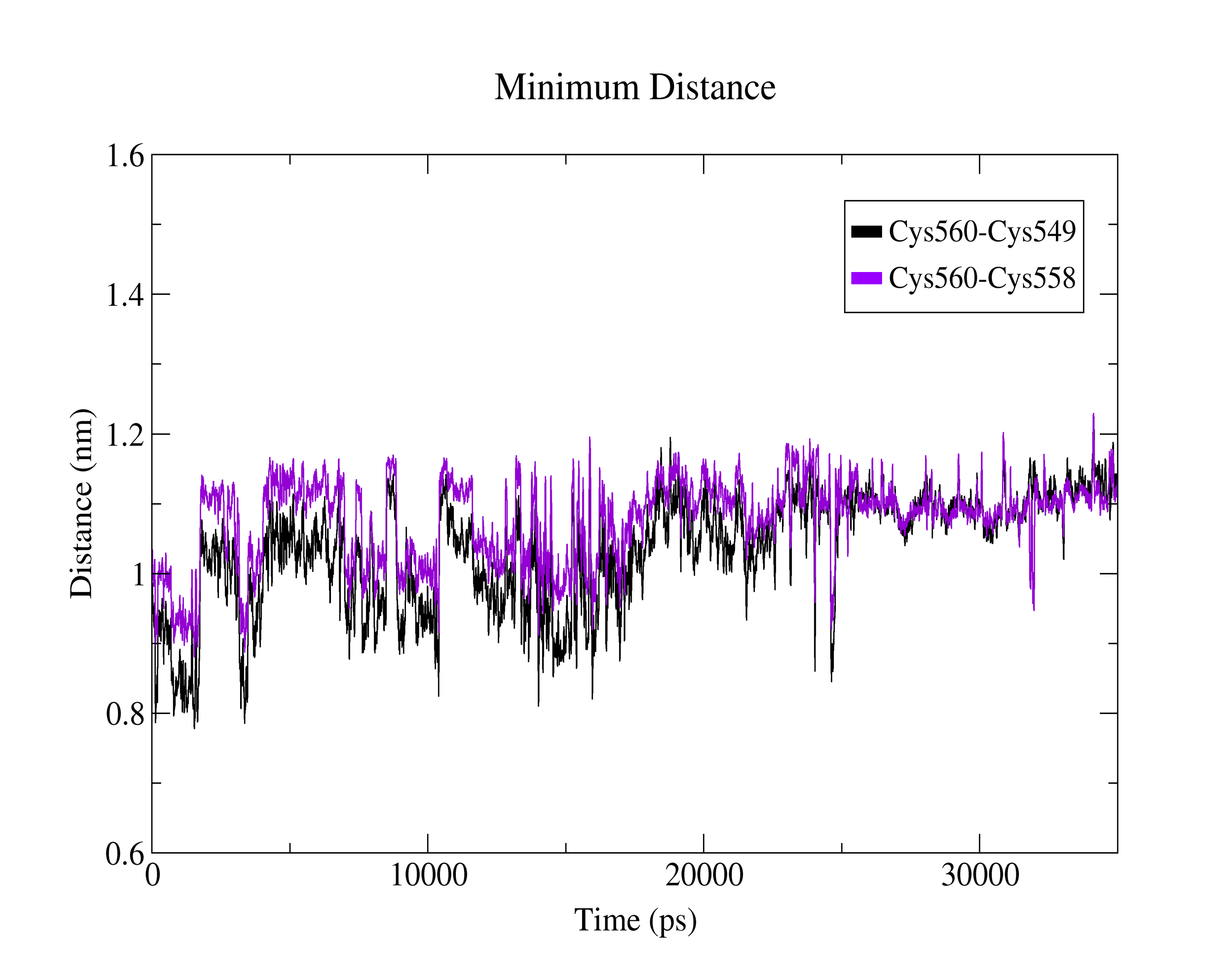

Supplement: Figure S3 — The minimum distance between the free thiol Cys560 (in the C583S simulation) and the disulfide pair Cys549–Cys558 in I-EGF3. The distance was calculated between the sulfur atoms. (TIF) [file pone.0059175.s003.tif]

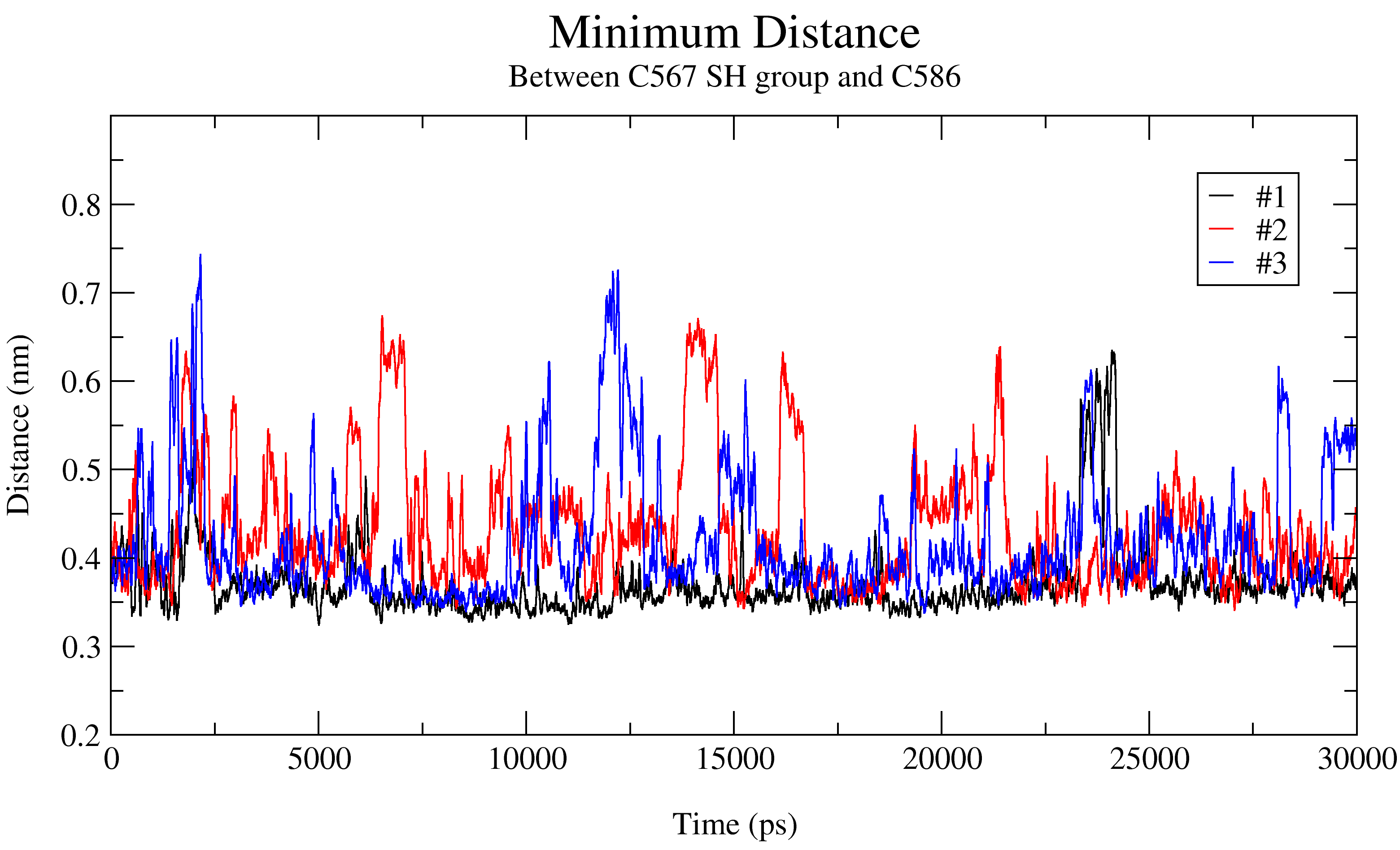

Supplement: Figure S4 — The minimum distance between the free thiol Cys567 and Cys586 in the three C581S simulations. The distance was calculated between the sulfur atoms. (TIF) [file pone.0059175.s004.tif]

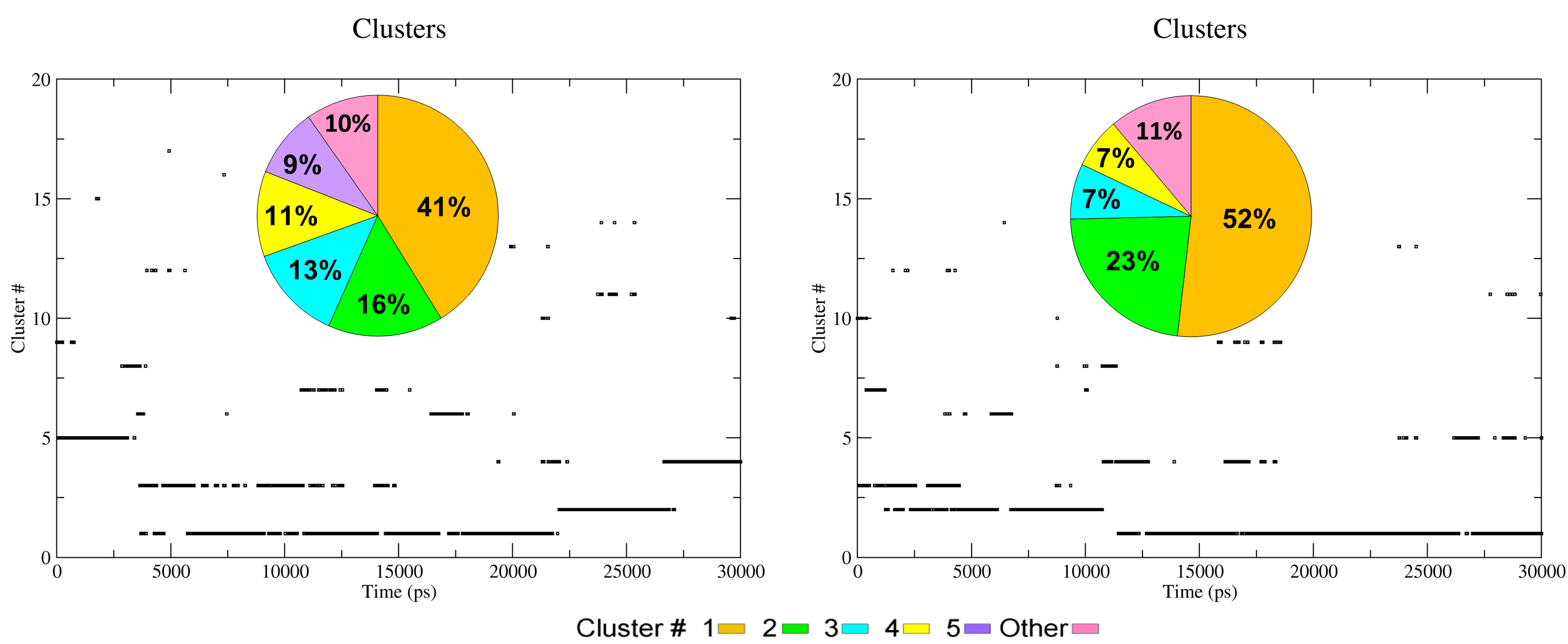

Supplement: Figure S5 — Cluster analysis of simulations number 2&3 of the C581S mutation (left & right respectively). The figure depicts the results by two presentations for the two simulations. The main frame shows the transitions between clusters (identified on the ordinate by numbers) along the trajectories. The relative fraction of the dominant clusters is presented by the colored histogram. The clusters are numbered sequentially from the most (#1) to the less popular. (TIF) [file pone.0059175.s005.tif]
