# Supplementary material for: A Single Disulfide Bond Disruption in the β3 Integrin Subunit Promotes Thiol/Disulfide Exchange, a Molecular Dynamics Study
Source: PLoS One. 2013 Mar 18;8(3):e59175. doi: 10.1371/journal.pone.0059175 (PMC3601072; doi:10.1371/journal.pone.0059175)
Supplement: Table S1 — List of C583S simulations. (DOCX) [file pone.0059175.s006.docx]

**Table S1:** List of C583S simulations

| **Simulation** | **Force Restraint** | **Number of atoms restrained** | **Simulation time (ns)** |
| --- | --- | --- | --- |
| 1 | 1000 kJ/mol*nm^2^ | 19^a^ | 48 |
|  |  |  |  |
| 2 | 1000 kJ/mol*nm^2^ | 19^a^ | 72 |
|  |  |  |  |
|  |  | After 40ns lowered to 3^b^ |  |
|  |  |  |  |
| 3 | 1000 kJ/mol*nm^2^ | 3^b^ | 53 |
| 4 | 1000 kJ/mol*nm^2^ | 3 ^b^ | 53 |
| 5 | 100 kJ/mol*nm^2^ | 3 ^b^ | 40 |
| 6 | 100 kJ/mol*nm^2^ | 3 ^b^ | 40 |
| 7 | 100 kJ/mol*nm^2^ | 3 ^b^ | 35 |

^a^ 1^st^ residue in the N terminal and 2 residues in the C terminal.

^b^ 1^st^ atom in the N terminal and 2 atoms in the C terminal.
